# Phylogenetic relationships, selective pressure and molecular markers development of six species in subfamily Polygonoideae based on complete chloroplast genomes

**DOI:** 10.1038/s41598-024-58934-7

**Published:** 2024-04-29

**Authors:** Zhan Feng, Yan Zheng, Yuan Jiang, Jin Pei, Linfang Huang

**Affiliations:** 1 Key Laboratory of Chinese Medicine Resources Conservation, State Administration of Traditional Chinese Medicine of the People’s Republic of China, Institute of Medicinal Plant Development, Chinese Academy of Medical Sciences & Peking Union Medical College, Beijing, 100193 China; 2 State Key Laboratory of Southwestern Chinese Medicine Resources, Chengdu University of Traditional Chinese Medicine, Chengdu, 611137 Sichuan China

**Keywords:** Chloroplast genome, Polygonoideae, Molecular marker, Phylogenetic analysis, Plant sciences, Plant molecular biology

## Abstract

The subfamily Polygonoideae encompasses a diverse array of medicinal and horticultural plants that hold significant economic value. However, due to the lack of a robust taxonomy based on phylogenetic relationships, the classification within this family is perplexing, and there is also a scarcity of reports on the chloroplast genomes of many plants falling under this classification. In this study, we conducted a comprehensive analysis by sequencing and characterizing the complete chloroplast genomes of six Polygonoideae plants, namely *Pteroxygonum denticulatum*, *Pleuropterus multiflorus*, *Pleuropterus ciliinervis*, *Fallopia aubertii*, *Fallopia dentatoalata*, and *Fallopia convolvulus*. Our findings revealed that these six plants possess chloroplast genomes with a typical quadripartite structure, averaging 162,931 bp in length. Comparative chloroplast analysis, codon usage analysis, and repetitive sequence analysis demonstrated a high level of conservation within the chloroplast genomes of these plants. Furthermore, phylogenetic analysis unveiled a distinct clade occupied by *P. denticulatum*, while *P. ciliinrvis* displayed a closer relationship to the three plants belonging to the *Fallopia* genus. Selective pressure analysis based on maximum likelihood trees showed that a total of 14 protein-coding genes exhibited positive selection, with *psbB* and *ycf1* having the highest number of positive amino acid sites. Additionally, we identified four molecular markers, namely *petN-psbM*, *psal-ycf4*, *ycf3-trnS-GGA*, and *trnL-UAG-ccsA*, which exhibit high variability and can be utilized for the identification of these six plants.

## Introduction

Subfamily Polygonoideae Eaton is one of the most species-rich subfamilies within the Polygonaceae family, containing plants from genera such as *Persicaria* (L.) Mill, *Polygonum* L, and *Fallopia* Adans^1^. Modern pharmacological studies have demonstrated that many plants from the Subfam. Polygonoideae possess significant medicinal value. For instance, *Pteroxygonum denticulatum* (C. C. Huang) T. M. Schust. & Reveal has anti-inflammatory, anti-gastric ulcer, and antibacterial properties^2^, while *Pleuropterus multiflorus* (Thunb.) Nakai exhibits hypolipidemic^3^ and antidiabetic effects^4^. These medicinal plants are primarily found in East Asia, specifically in China, Japan, and Korea, and have a long history of use in traditional Chinese medicine.

Compared to other plant families, identification of Polygonaceae is generally straightforward. However, distinguishing them within the family itself poses challenges due to significant morphological variations. Consequently, taxonomic arrangements have been subject to discrepancies and the proliferation of names^5^. The tribe Persicarieae Dumort., belonging to the Subfam. Polygonoideae, has encountered such issues. For instance, the 1998 edition of FRPS (Flora Reipublicae Popularis Sinicae) reported approximately 20 species within the genus *Fallopia*, including *Fallopia multiflora* (synonymous with *Pleuropterus multiflorus*) and *Fallopia denticulata* (synonymous with *Pteroxygonum denticulatum*)^1^. In 2015, Schuster et al.^6^ screened three barcodes (*nrITS*, *matK*, *trnL-trnF*) and analyzed 199 species of Polygonaceae using maximum likelihood and Bayesian methods. Their results showed that *P. denticulatum* clustered with *Pteroxygonum giraldii* Damm. et Diels as a branch and that *Fallopia denticulata* (Huang) A. J. Li was revised to *Pteroxygonum denticulatum* (C. C. Huang) T. M. Schust. & Reveal. Furthermore, the “Catalogue of Life China: 2023 Annual Checklist” reassigned Genus *Fallopia*, as documented in the Flora of China, into three genera: *Pteroxygonum* (including *P. denticulatum* and *P. giraldii*), *Pleuropterus* (including *P. multiflorus* and *Pleuropterus ciliinervis* Nakai), and *Fallopia* (including *Fallopia aubertii* (L. Henry) Holub, *Fallopia convolvulus* (L.) Á. Löve, *Fallopia cynanchoides* (Hemsl.) Harald., *Fallopia dentatoalata* (F. Schmidt) Holub, and *Fallopia dumetorum* (Linnaeus) Holub)^7^. Although the classification of Polygonaceae plants is continuously being updated, the latest plant revision names have not been widely promoted. For instance, most recent studies on *P. multiflorus* still use its synonym *F. multiflora*^8–10^.

Chloroplasts play a crucial role in energy conversion and photosynthesis in plants. They are also involved in developmental processes, secondary metabolic activities^11^, and facilitate gene expression coordination between organelles and the nuclear genome^12^. Chloroplasts are found in land plants, algae, and certain protozoa. In plants, the chloroplast genome constitutes one of the three genetic systems, alongside the nucleus and mitochondria. These genetic systems contain both eukaryotic introns and prokaryotic operons^13^. Furthermore, chloroplasts are semi-autonomous organelles, possessing their own transcription and transport systems, as well as independent genomes^14^. In addition to synthesizing sugars through photosynthesis, chloroplasts also participate in the synthesis of complex organic substances such as amino acids and fatty acids^15^.

The advancement of high-throughput sequencing technologies has significantly enhanced our understanding of chloroplast genomes. Modern chloroplast genomes exhibit common structural features, typically ranging in size from 107 to 218 kb, characterized by compactness and containing approximately 100–120 genes^16^. A typical chloroplast genome follows a cyclic pattern and consists of four components: a large single-copy (LSC) region, a small single-copy (SSC) region, and two inverted repeat (IR) regions^17,18^. The variation in chloroplast genomes among different plant species mainly stems from the contraction and expansion of the IR region^19,20^. Chloroplast genomes exhibit high conservation in terms of organization, gene order, and content, ensuring homology across evolutionary groups. Moreover, these genomes are effectively haploid, ensuring genetic homogeneity among species. As a result, chloroplast genomes serve as an ideal model for investigating species identification and evolution. For instance, Yu and Ye^21,22^ conducted sequencing of the chloroplast genomes of *Polygonum chinense* L. and *Polygonum cuspidatum* Siebold et Zucc., classifying the plants based on the obtained results. Guo et al.^23^ sequenced and assembled the chloroplast genomes of four species from genus *Polygonum*, followed by phylogenetic analysis, revealing the relationships between them. Additionally, Chen et al.^24^ analyzed chloroplast genomes of *Reynoutria japonica* Houtt. from different regions, performing phylogenetic analyses that highlighted *R. japonica*'s close association with *P. multiflorus*. Furthermore, Chinese *R. japonica* could be further divided into two distinct main groups.

Chloroplast genomes offer a valuable genetic resource for investigating genetic and evolutionary relationships among species in the Polygonaceae family. However, studies focusing on chloroplast genomes of Polygonaceae species remain limited. To address this knowledge gap, this study pursued the following objectives: (i) sequencing and assembling the chloroplast genomes of six plants from Subfam. Polygonoideae (*P. denticulatum*, *P. multiflorus*, *P. ciliinervis*, *F. aubertii*, *F. dentatoalata*, *F. convolvulus*) to contribute to the field of chloroplast research, (ii) reconstructing the phylogeny by integrating the sequencing results with available chloroplast genomes of other published Polygonaceae plants to provide robust evidence for the classification of species from Subfam. Polygonoideae, and (iii) developing molecular markers based on highly variable regions within the chloroplast genome.

## Materials and methods

### Sample collection, DNA extraction, and sequencing

**Figure 1:**
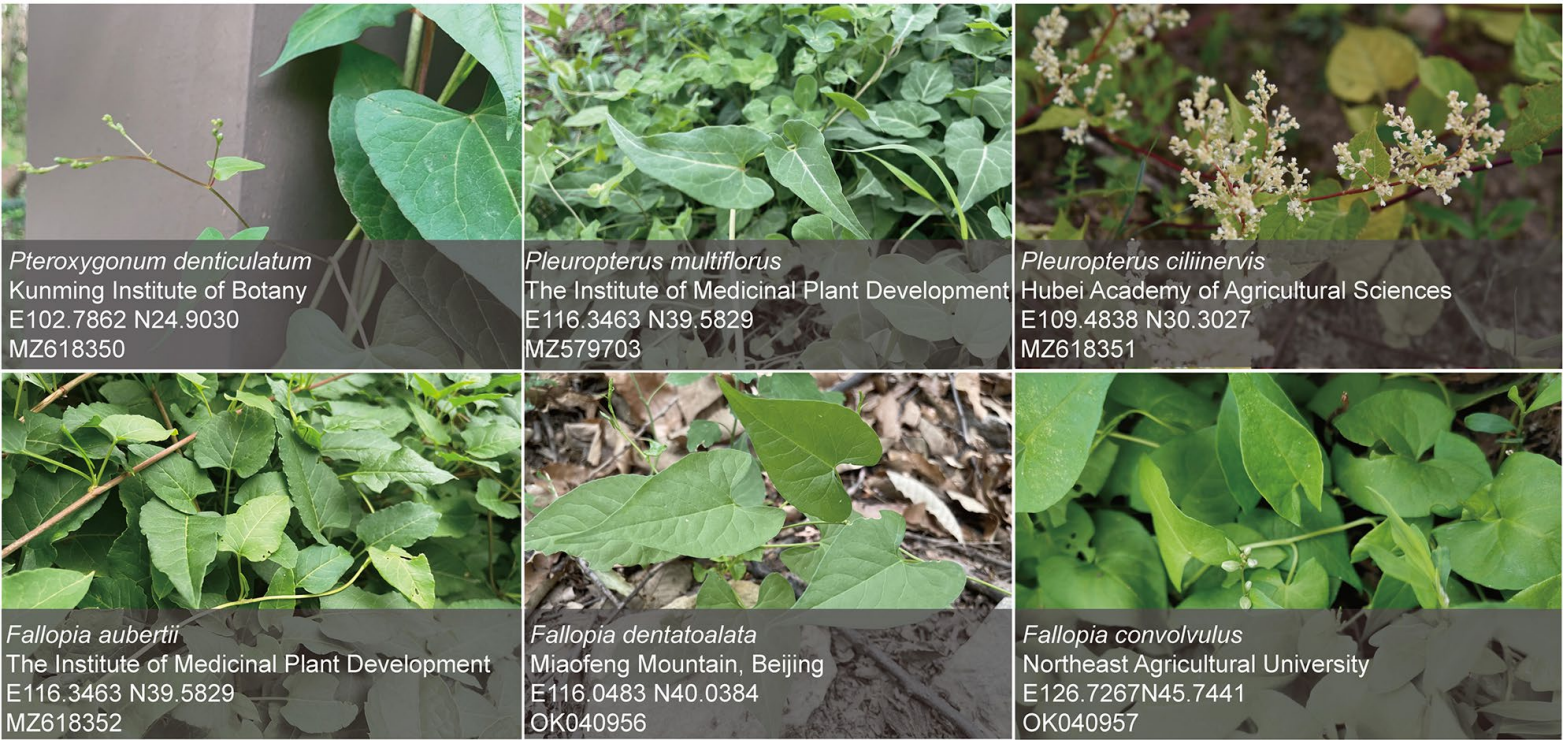
Fresh leaf collection sites of six species of Subfam. Polygonoideae.

The collection of fresh leaves from the six Subfam. Polygonoideae species was conducted in accordance with the IUCN Policy Statement on Research Involving Species at Risk of Extinction and the Convention on the Trade in Endangered Species of Wild Fauna and Flora, with permission obtained from the local authorities at the collection sites (Fig. 1). The samples were identified as *Pteroxygonum denticulatum* (C. C. Huang) T. M. Schust. & Reveal, *Pleuropterus multiflorus* (Thunb.) Nakai, *Pleuropterus ciliinervis* Nakai, *Fallopia aubertii* (L. Henry) Holub, *Fallopia dentatoalata* (F. Schmidt) Holub and *Fallopia convolvulus* (L.) Á. Löve by Professor Yu-Lin Lin of the Institute of Medicinal Plants, Chinese Academy of Medical Sciences, and voucher specimens were stored in the Herbarium of the same institute with the codes CMPB57201, CMPB57202, CMPB57203, CMPB57204, CMPB57205 and CMPB57206. All fresh leaves underwent thorough washing with distilled water, followed by drying. The dried leaves were then carefully wrapped in tin foil, labeled, and rapidly frozen in liquid nitrogen. Subsequently, the samples were placed in a transport box filled with dry ice to maintain their frozen state. To ensure preservation, the samples were stored at −80 °C in an ultra-low temperature refrigerator, facilitating subsequent DNA extraction and other experimental studies.

The extraction of plant genome utilized the Plant Genome DNA Extraction Kit (Tiangen Biochemical Technology (Beijing) Co., Ltd.). Subsequently, the DNA's quality and concentration were assessed through 1% (w/v) agarose gel electrophoresis and Qubit 3.0 (Thermo Fisher Scientific, USA). For sequencing, the Illumina technology platform was employed, and a library with a 400 base pairs (bp) insert size was constructed using 500 ng of DNA. The sequencing process followed a double-end (2 × 150 bp) sequencing strategy as per the manufacturer's instructions for the Hiseq platform (Illumina Inc, USA).

### Chloroplast genome assembly and annotation

The clean data was assembled on a Linux operating system using the Get Organelle software^25^. To validate the accurate assembly of chloroplast genes, raw reads were mapped to the clean data using BOWtie2 software (v2.0.1)^26^. This mapping process assessed the coverage of chloroplast genome sequences and confirmed the integrity of individual contig junctions. Following assembly, the six species were subjected to annotation using the CPGAVAS2 online platform^27^, with *P. multiflorus* (NC_041239) serving as the reference. Manual adjustment and correction of gene boundary positions were performed using the Apollo software^28^. Visualization of the annotated results was accomplished through the CHLOROPLOT (https://irscope.shinyapps.io/Chloroplot/) online platform^29^.

### Genome comparison

The fundamental characteristics of the assembled and annotated chloroplast genomes of the six plant species were examined. Geneious v11.1 (https://www.geneious.com) was employed to determine the total length, gene count, base content, gene length, and repetitive gene count for each species' chloroplast genomes. The Blast tool^30^ and the "Find Repeats regions" script were utilized to identify the large single copy (LSC) and small single copy (SSC) regions within the plant chloroplast genome, along with the two inverted repeats that separate them. Through a comparison of the tetrameric regions' genome lengths and base contents, the total genome lengths and base contents of the six plants were derived. Furthermore, the genes annotated in the genomes of each species were categorized based on their functional classification, and the differences in functional genes were quantified.

To assess differences in the boundaries of the four regions (IRa, IRb, LSC, SSC) within the chloroplast genome of the six species, the online visualization tool IRscope^31^ was utilized. The occurrence of gene rearrangement was examined using Mauve^32^, and collinearity analysis was conducted on the chloroplast genomes of the six species. Furthermore, a genome-wide comparative analysis was performed utilizing the mVISTA (http://genome.lbl.gov/vista/mvista/submit.shtml) online platform^33,34^ to explore discrepancies among the six sequences.

### Analysis of base composition and codon usage

The base composition differences and the relative usage of synonymous codons among the six chloroplast genomes were examined. For this analysis, the Geneious v11.1 was employed to determine the base composition of the six chloroplast genome sequences. Furthermore, all protein-coding sequences were extracted, exported to fasta format, and consolidated into a single fasta file, using Geneious v11.1 software. To assess the distribution of codon usage, the CodonW with relative synonymous codon usage (RSCU) ratios was utilized for the final analysis.

### Repeated Sequence and Simple Sequence Repeat Analysis

The identification of long fragment repeats within the chloroplast genome sequences was performed using the online tool REPuter^35^. The parameters were configured as follows: Forward repeat, Reverse repeat, Complement repeat, and Palindrome repeat. The minimum length for a repeat unit was set to 30 bp, and the maximum number of detected repeat sequences was limited to 1000. The similarity threshold for repeat sequences was set at 90%, while the Hamming distance was specified as 3.

The detection of simple sequence repeats (SSRs) sequences within the chloroplast genome sequences was accomplished using the MISA script, developed in the Perl programming language^36^. The parameter settings were as follows: mononucleotide repeats with a minimum of 10 repeats, dinucleotide repeats with a minimum of 8 repeats, trinucleotide repeats with a minimum of 4 repeats, and tetranucleotide, pentanucleotide, and hexanucleotide repeats with a minimum of 3 repeats^37^.

### Phylogenetic analysis

To investigate the phylogeny of Polygonaceae, a phylogenetic analysis was conducted using the chloroplast genomes of 29 species from the family Polygonaceae and two outgroups (*Myricaria prostrata* NC_046761.1, *Tamarix taklamakanensis* NC_054218.1). A total of 23 sequences were obtained from the NCBI database^38^, while the remaining six sequences were obtained through sequencing in this study. The phylogenetic tree was constructed using the Maximum Likelihood (ML), Maximum Parsimony (MP), and Neighbor-Joining (NJ) methods. The coding sequences (CDS) of the entire genome and the full-length chloroplast sequences were separately utilized for the tree construction.

The CDS from the chloroplast genome sequences of 31 species were extracted using the "Extract" function in PhyloSuite^39^. The sequence type was set to "Chloroplast Genome". Multi-copy genes were screened, and the selected coding sequences were saved in fasta format. The "MAFFT" function^40^ in PhyloSuite was utilized to perform multiple gene alignment of the CDS sequences. The alignment mode was set to "Normal", with the standard code and an auto strategy (-auto). The aligned CDS sequences were then concatenated using the "Concatenate Sequence" function in PhyloSuite. The resulting concatenated sequences were saved in fasta, phy, and nex formats. For constructing the ML tree, the "IQ-TREE" function in PhyloSuite^41^ was employed. The concatenated phy format file was imported, with *M. prostrata* and *T. taklamakanensis* selected as the outgroup. The default parameters were utilized. To build the MP trees, the "Construct/Test Maximum Parsimony Tree(s)" function in MEGA-X software^42^ was utilized. The concatenated fasta file obtained earlier was imported, selecting "Nucleotide Sequences" as the data type and "Standard" as the gene model. The phylogenetic test was performed using the bootstrap method with 1000 replicates, while the remaining parameters were set to their default values. The NJ tree was constructed using the "Construct/Test Neighbor-Joining Tree(s)" function in MEGA-X software^42^. The concatenated fasta format file was imported, selecting "Nucleotide sequence" as the data type and "Standard" as the gene mode. The phylogeny test was conducted using the bootstrap method with 1000 replicates, and the model/method selected was "p-distance". The default values were used for the remaining parameters.

### Non-synonymous and synonymous substitution rate analysis

To identify the genes under selection, we scanned the chloroplast genomes of 29 species from the family Polygonaceae and two outgroups (*Myricaria prostrata* NC_046761.1, *Tamarix taklamakanensis* NC_054218.1) using the software EasyCondeML^43^. This tool facilitated the computation of non-synonymous (dN) and synonymous (dS) substitution rates, as well as their ratios (ω = dN/dS). Selective pressure analyses were carried out on the maximum likelihood (ML) tree of these 31 species, formatted in Newick. Each single-copy CDS sequence was aligned based on its amino acid sequence. We employed a site-specific model with five site models (M0, M1a & M2a, M7 & M8) to pinpoint adaptation signatures across chloroplast genomes. This model allowed the ω ratio to vary among sites while maintaining a fixed ω ratio across all branches. Specifically, the site-specific models, M1a (nearly neutral) vs. M2a (positive selection), and M7 (β) vs. M8 (β & ω) were computed to detect positive selection^44^. Likelihood ratio tests (LRT) comparing M1a vs. M2a and M7 vs. M8 were employed to assess the strength of selection. Subsequently, the Bayes empirical Bayes (BEB) method^45^ was utilized to calculate posterior probabilities. In the BEB analysis, posterior probabilities exceeding 0.95 and 0.99 indicated sites subject to positive selection and strong positive selection, respectively.

### Analysis of highly variable areas

A custom script was developed to identify the most divergent regions. Using this script, the intergenic spacer regions (IGS) of six plant chloroplasts were extracted from the GenBank file, with their respective starting and ending points. The extracted sequences were then compared using the ClustalW2 program (v. 2.0.12) with the parameters "-type = DNA -gapopen = 10 -gapext = 2"^46^. Pairwise distances were calculated using the K2p evolutionary model implemented in the distmat program of the EMBOSS package (v. 6.3.1)^47^.

### Primer design and PCR validation

To facilitate the development of molecular markers for the six plants, we screened the highly variable regions within their chloroplast genomes. The selected candidate sequences had a length ranging from 300 to 1000 bp, ensuring a high success rate for amplification and sequencing. Moreover, to facilitate the design of universal primers, the candidate sequences were conserved at both ends. Subsequently, the primers were designed using SnapGene software, and the primer sequences were submitted to TsingkeBiotechnology Co, Ltd.

We carefully selected primers that displayed clear, single, and accurate amplification bands across individual samples. Subsequently, PCR amplification was conducted on the total DNA extracted from each of the six plants. The reaction protocol involved an initial denaturation step at 95 °C for 3 min, followed by 32–36 cycles consisting of denaturation at 94 °C for 25 s, annealing at 55–64 °C for 25 s, and extension at 72 °C for 10–15 s. A final extension step was performed at 72 °C for 5 min. The resulting PCR amplification products were visualized by gel electrophoresis using a 1.5% agarose gel stained with SYBR. Gel electrophoresis was carried out under the following conditions: 130 V, 120 mA, 300 W for 45 min. Subsequently, the PCR amplification products were sent to TsingkeBiotechnology Co, Ltd. for further processing. The sequencing peak maps obtained from the sequencing company were analyzed using SnapGene software. The software was used to read the peak maps, perform proofreading, and eliminate low-quality sequences as well as primer regions.

## Result

### Comparative analysis of chloroplast genomes of six species of Polygonaceae

Table S1 presents the data acquired through sequencing on the Illumina HiSeq platform. The largest total raw read count was 21,319,251 bp for *F. aubertii*, while the smallest count was 17,329,101 bp for *P. ciliinervis*.

#### Chloroplast genome sequence characteristics and gene structure

**Figure 2:**
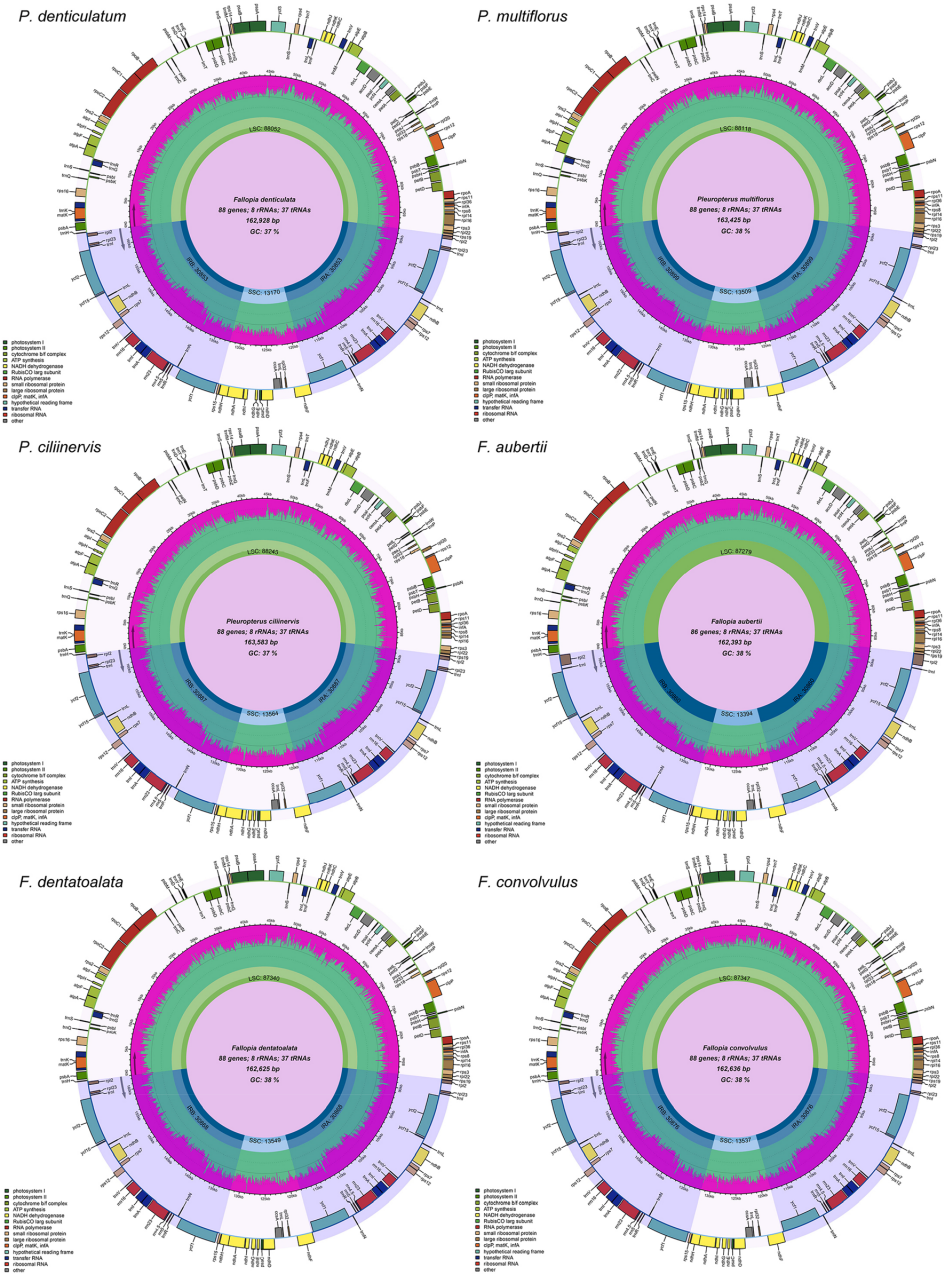
The complete chloroplast genome map of six species of Subfam. Polygonoideae.

The chloroplast genomes of the six plants analyzed in this study exhibit a typical quadripartite structure consisting of LSC region, SSC region, and two IR regions (Fig. 2). Among the plants studied, *P. ciliinervis* has the longest chloroplast genome, measuring 163,583 bp, while *F. aubertii* has the shortest chloroplast genome, measuring 162,393 bp. The average length of the chloroplast genomes in the six studied plants was 162,931 bp, and their differences in length were minimal, with the largest discrepancy being only 1,190 bp. Regarding the LSC, SSC, and IR regions, the LSC region spanned from 87,279 to 88,245 bp, with an average length of 87,730 bp. The SSC region ranged from 13,170 to 13,564 bp, with an average length of 13,453 bp. Finally, the IR region varied from 30,853 to 30,899 bp, with an average length of 30,873 bp.

In the complete chloroplast genome, the GC content ranged from 37.38% to 37.66%. Furthermore, the LSC, SSC, and IR regions exhibited GC contents of 35.39–35.83%, 32.28–32.92%, and 41.21–41.28%, respectively (Table S2). Table 1 provide a comprehensive overview of the chloroplast genomes of these six plants, revealing a total of 133 genes encoded, including 88 protein-coding genes, 37 (Transfer RNA) tRNA genes, and 8 rRNA (Ribosome RNA) genes. Notably, each IR region contained 16 genes, encompassing 4 rRNA genes, 6 tRNA genes, and 6 protein-coding genes. The chloroplast genomes of the six plants analyzed in this study primarily consisted of genes associated with photosynthesis and self-replication. The photosynthetic gene category encompassed subunits of ATP synthase, subunits of photosystem II, and subunits of NADH-dehydrogenase, among others. The self-replicating gene category included the large subunit of ribosome, DNA-dependent RNA polymerase, and small subunit of ribosome. *P. denticulatum* and *F. aubertii* each possess 21 genes with introns, while *P. multiflorus*, *F. dentatoalata*, and *F. convolvulus* have 19 genes with introns, and *P. ciliinervis* has 18 genes with introns. Notably, all these plants share two identical genes, *clpP* and *ycf3*, each containing two introns and three exons (Table S3).

**Table 1:** Gene composition and gene function of chloroplast genomes of six species.

Composition and function	Groups of genes	Gene Name
Composition of genes	Protein coding gene	*accD*, *atpA*, *atpB*, *atpE*, *atpF*, *atpH*, *atpI*, *ccsA*, *cemA*, *clpP*, *infA*, *matK*, *ndhA*, *ndhB* (×2), *ndhC*, *ndhD*, *ndhE*, *ndhF*, *ndhG, ndhH*, *ndhI*, *ndhJ*, *ndhK*, *petA*, *petB*, *petD*, *petG*, *petL*, *petN*, *psaA*, *psaB*, *psaC*, *psaI*, *psaJ*, *psbA*, *psbB*, *psbC*, *psbD*, *psbE, psbF*, *psbH*, *psbI*, *psbJ*, *psbK*, *psbM*, *psbN*, *psbT*, *psbZ*, *rbcL*, *rpl14*, *rpl16*, *rpl2* (×2), *rpl20*, *rpl22*, *rpl23* (×2), *rpl32*, *rpl33*, *rpl36*, *rpoA*, *rpoB*, *rpoC1*, *rpoC2*, *rps11*, *rps12* (×2), *rps14*, *rps15*, *rps16*, *rps18*, *rps19*, *rps2*, *rps3*, *rps4*, *rps7* (×2), *rps8*, *ycf1* (×2), *ycf2* (×2), *ycf3*, *ycf4*, *ycf15* (×2)
	Transfer RNA genes	*trnA-UGC* (×2), *trnC-GCA*, *trnD-GUC, trnE-UUC*, *trnF-GAA, trnfM-CAU*, *trnG-GCC*, *trnG-UCC*, *trnH-GUG*, *trnI-CAU* (×2), *trnI-GAU* (×2), *trnK-UUU*, *trnL-CAA* (×2), *trnL-UAA*, *trnL-UAG*, *trnM-CAU*, *trnN-GUU* (×2), *trnP-UGG*, *trnQ-UUG*, *trnR-ACG* (×2), *trnR-UCU*, *trnS-GCU*, *trnS-GGA*, *trnS-UGA*, *trnT-GGU*, *trnT-UGU*, *trnV-GAC* (×2), *trnV-UAC*, *trnW-CCA*, *trnY-GUA*
	Ribosomal RNA genes	*rrn16S* (×2), *rrn23S* (×2), *rrn4.5S* (×2), *rrn5S* (×2)
Genes for photosynthesis	Subunits of ATP synthase	*atpA, atpB, atpE, atpF, atpH, atpI*
	Subunits of photosystem II	*psbA, psbB, psbC, psbD, psbE, psbF, psbI, psbJ, psbK, psbM, psbN, psbT, psbZ, ycf3*
	Subunits of NADH-dehydrogenase	*ndhA, ndhB* (×2)*, ndhC, ndhD, ndhE, ndhF, ndhG, ndhH, ndhI, ndhJ, ndhK*
	Subunits of cytochrome b/f complex	*petA, petB,* petD*, petG, etL, petN*
	Subunits of photosystem I	*psaA, psaB, psaC, psaI, psaJ*
	Subunit of rubisco rbcL	*rbcL*
Self replication genes	Large subunit of ribosome	*rpl14, rpl16, rpl2* (×2)*, rpl20, rpl22, rpl23* (×2)*, rpl32, rpl33, rpl36*
	DNA dependent RNA polymerase	*rpoA, rpoB, rpoC1, rpoC2*
	Small subunit of ribosome	*rps11, rps12* (×2)*, rps14, rps15, rps16, rps18, rps19, rps2, rps3, rps4, rps7* (×2)*, rps8*
Other genes	Subunit of Acetyl-CoA-carboxylase	*accD*
	c-type cytochrom synthesis gene	*ccsA*
	Envelop membrane protein	*cemA*
	Protease	*clpP*
	Translational initiation factor	*infA*
	Maturase	*matK*
Unkown	Conserved open reading frames	*ycf1* (×2) (×2)*, ycf15* (×2)*, ycf2* (×2)*, ycf4*

Note: Genes outside the circle are transcribed counterclockwise, while genes inside the circle are transcribed clockwise. Genes with different functions are indicated by different colors. The purple inner circle indicates GC content, while the green inner circle indicates AT content.

#### Comparative IR/SC boundary analysis

**Figure 3:**
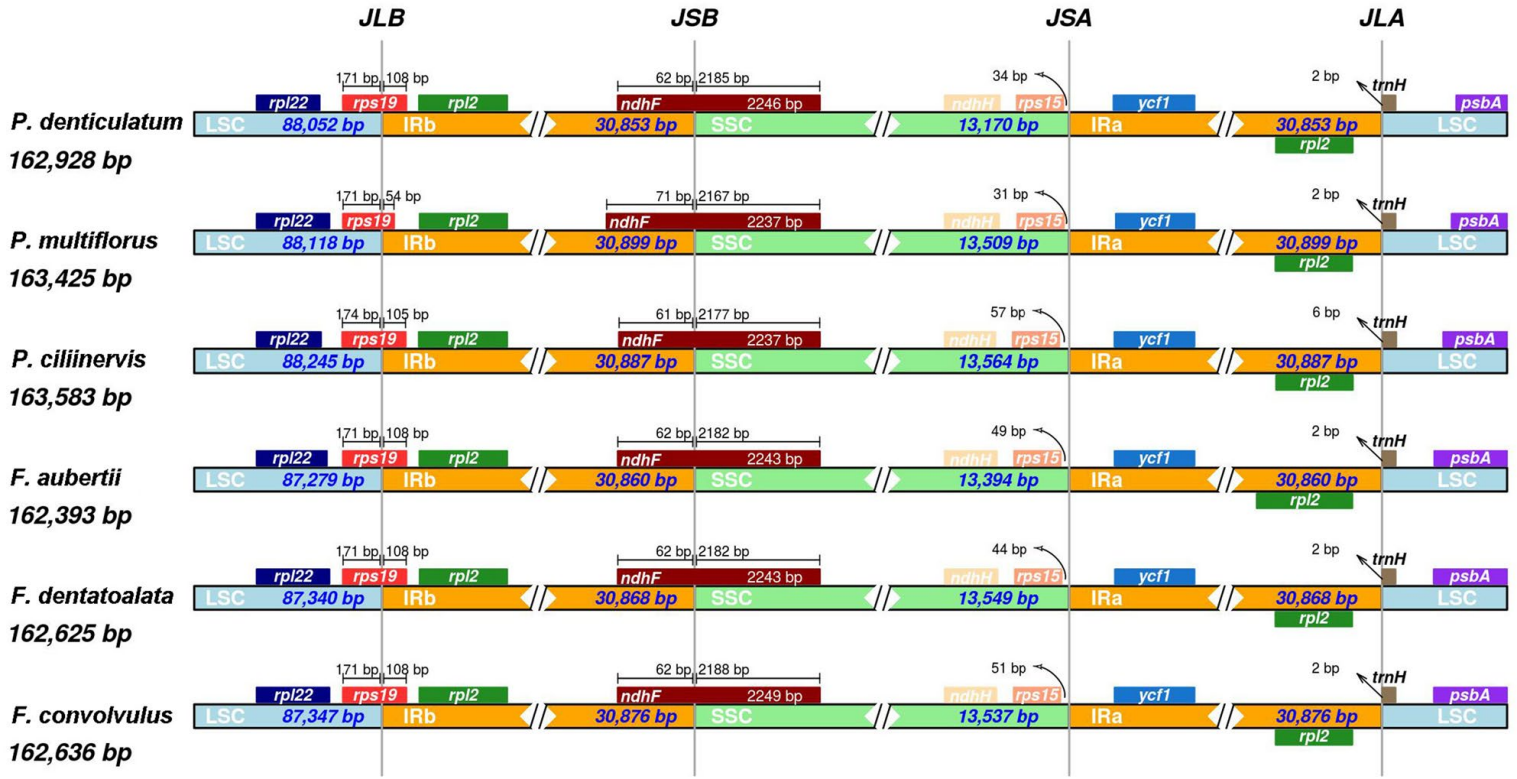
Comparison of LSC, SSC and IR region boundaries in six plants of Subfam. Polygonoideae.

In this study, we conducted a comparative analysis of expansion and gene rearrangement at the boundaries of the LSC, SSC, and IR regions in the chloroplast genomes of six species (Fig. 3). Each of the six species possessed a complete LSC region spanning from 87,279 bp to 88,245 bp, an SSC region ranging from 13,170 bp to 13,564 bp, and an IR region in between spanning from 30,853 bp to 30,899 bp. Due to differential expansion and contraction of the LSC/IRb and IRb/SSC regions within the chloroplast genome of these six plants, significant alterations occurred at the boundaries of this region. For instance, the IRa region exhibited loss of the *rps19* and *ndhF*, while IRb region exhibited loss of the *ycf1*. The results indicated that the distribution and length of genes at the boundary of the chloroplast quadripartite structure were highly consistent among the six plants, yet each underwent varying degrees of expansion and contraction.

#### Collinearity analysis and sequence variation analysis of the chloroplast genome

**Figure 4:**
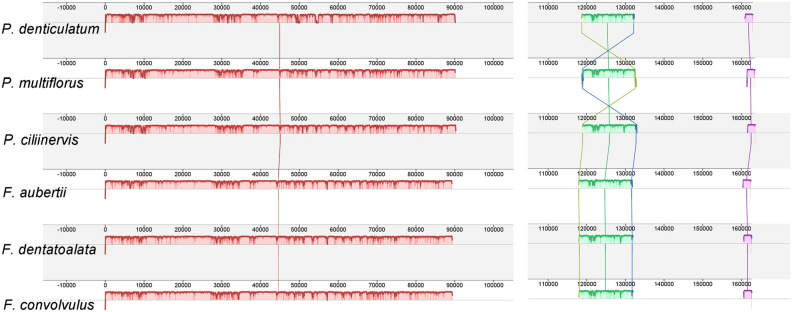
Collinearity analysis of six chloroplast genomes of Subfam. Polygonoideae using Mauve (with *P. multiflorus* as reference).

**Figure 5:**
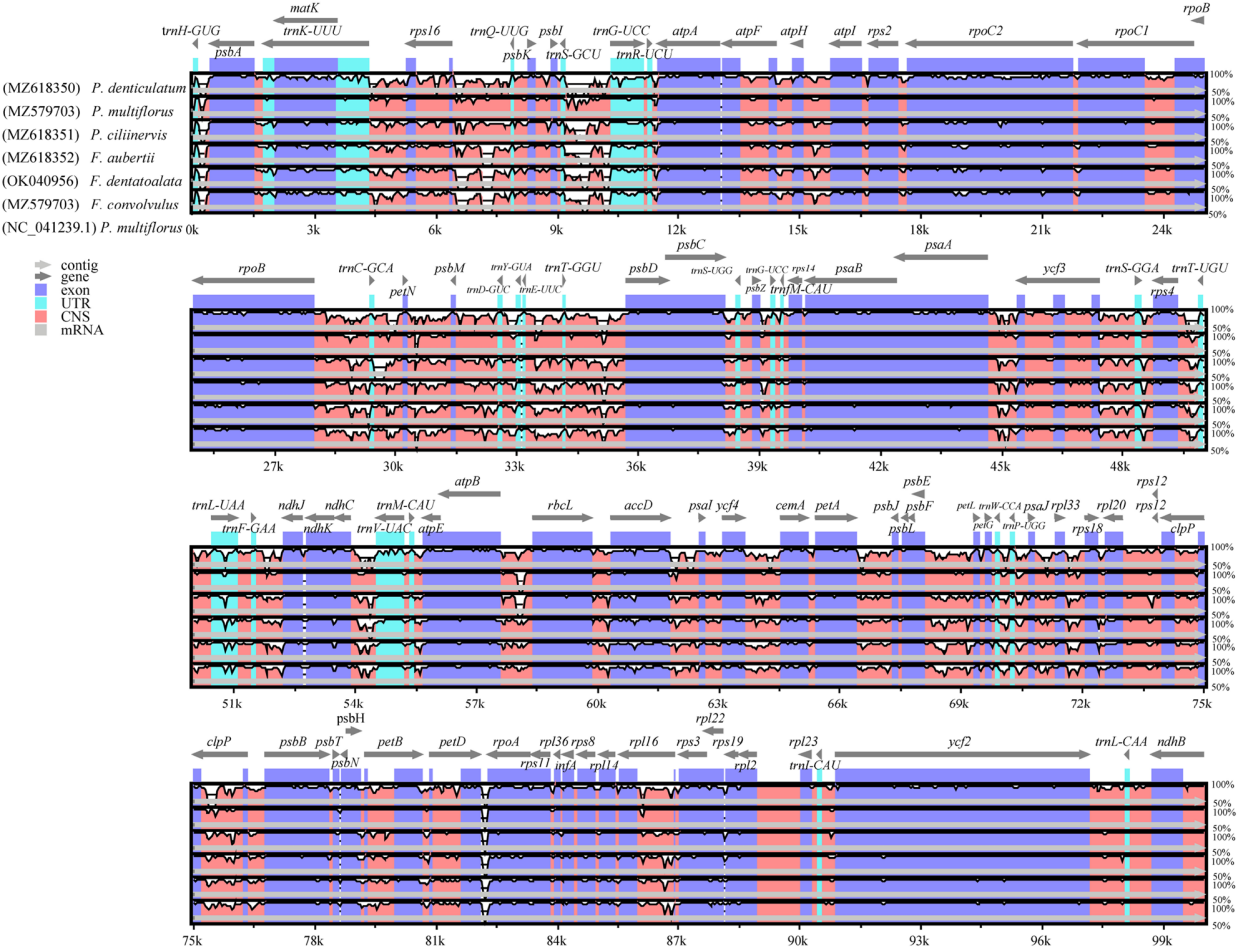
Visual analysis of six plants of Subfam. Polygonoideae of chloroplast genomes using mVISTA (LAGAN mode).

Note: Gray arrows represent genes, purple areas represent exon regions, blue areas represent tRNA regions, red areas represent IGS and intron regions, brown areas represent rRNA regions, vertical scale represents the percentage of identity, ranging from 50 to 100%.

The chloroplast genomes of the six species were subjected to collinearity analysis using Mauve software, revealing that gene rearrangements were absent in these plants' chloroplast genomes (Fig. 4). To further elucidate the differences among the chloroplast sequences of the six species, a global sequence alignment was performed using mVISTA. The results revealed minimal variations among the chloroplast sequences of the six species, indicating a high degree of sequence conservation (Fig. 5). Nonetheless, several regions and genes, namely *rps16* ~ *trnQ-UUG*, *trnS-GCU* ~ *trnG-UCC*, *rpoB* ~ *trnC-GCA*, *petN* ~ *psbM*, *rps4* ~ *trnT-UGU*, *petD* ~ *rpoA*, and *rpl16*, exhibited notably higher variation rates within the six chloroplast genomes. These regions and genes hold substantial potential for supporting future studies on phylogeny, genetic diversity, and species identification.

### Analysis of codon usage

**Figure 6:**
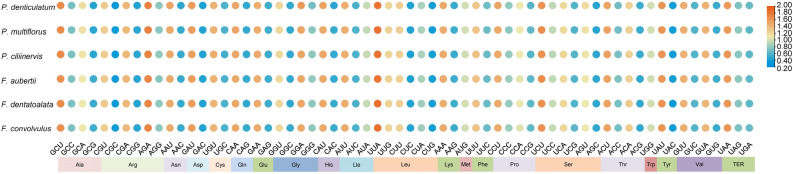
Codon content of 20 amino acids and stop codons in the protein-coding genes of the chloroplast genomes of six species of Subfam. Polygonoideae.

The codon usage frequencies of the six chloroplast genomes, comprising 27,413 to 27,676 codons (Fig. 6). The most commonly utilized amino acids were arginine (Arg) and leucine (Leu). Conversely, amino acids such as cysteine (Cys), aspartic acid (Asp), histidine (His), methionine (Met), proline (Pro), glutamine (Gln), arginine (Arg), tryptophan (Trp), and tyrosine (Tyr) had less than 2,000 codons. Notably, cysteine (Cys) exhibited the lowest frequency, occurring only 603 times, accounting for 1.29% of the total codons. In terms of stop codons, all six species predominantly employed UAA, which accounted for over 50% of occurrences. Thirty codons in all six species possessed RSCU values greater than 1, with 29 of them ending in A/U, except for UUG encoding leucine (Leu), which deviated from the A/U pattern. On the other hand, 32 codons had RSCU values below 1, with 29 of them ending in C/G, except for AUA for isoleucine (Ile), CUA for leucine (Leu), and UGA for the terminator (TER), which did not end in C/G. Methionine (Met) and tryptophan (Trp) displayed an RSCU value of 1, signifying no particular preference in codon usage.

### Repeated sequence and simple sequence repeat analysis

Table 2 reveals that *P. denticulatum* exhibited the highest number of SSRs, amounting to 62. Among these, the mononucleotide SSRs were predominantly composed of thymine (T) and adenine (A) repeats, while the dinucleotide SSRs primarily consisted of TA repeats. Notably, the guanine (G) repeat type was only detected in *F. convolvulus*. Upon further examination and comparison of the size and position of the different SSR units, it was found that T and A repeats were the most abundant SSR types across all six species.

**Table 2:** SSR analysis of chloroplast genomes of six species of Subfam. Polygonoideae.

*P. denticulatum*		*P. multiflorus*		*P. ciliinervis*		*F. aubertii*		*F. dentatoalata*		*F. convolvulus*	
Types	Number	Types	Number	Types	Number	Types	Number	Type	Number	Types	Number
A	22	A	22	A	22	A	15	A	14	A	18
C	1	C	–	C	1	C	–	C	–	C	–
G	–	G	–	G	–	G	–	G	–	G	1
T	32	T	22	T	18	T	16	T	16	T	14
AT	2	AT	–	AT	2	AT	4	AT	5	AT	8
TA	5	TA	4	TA	1	TA	2	TA	2	TA	3
Total	62	Total	53	Total	44	Total	37	Total	37	Total	44

**Figure 7:**
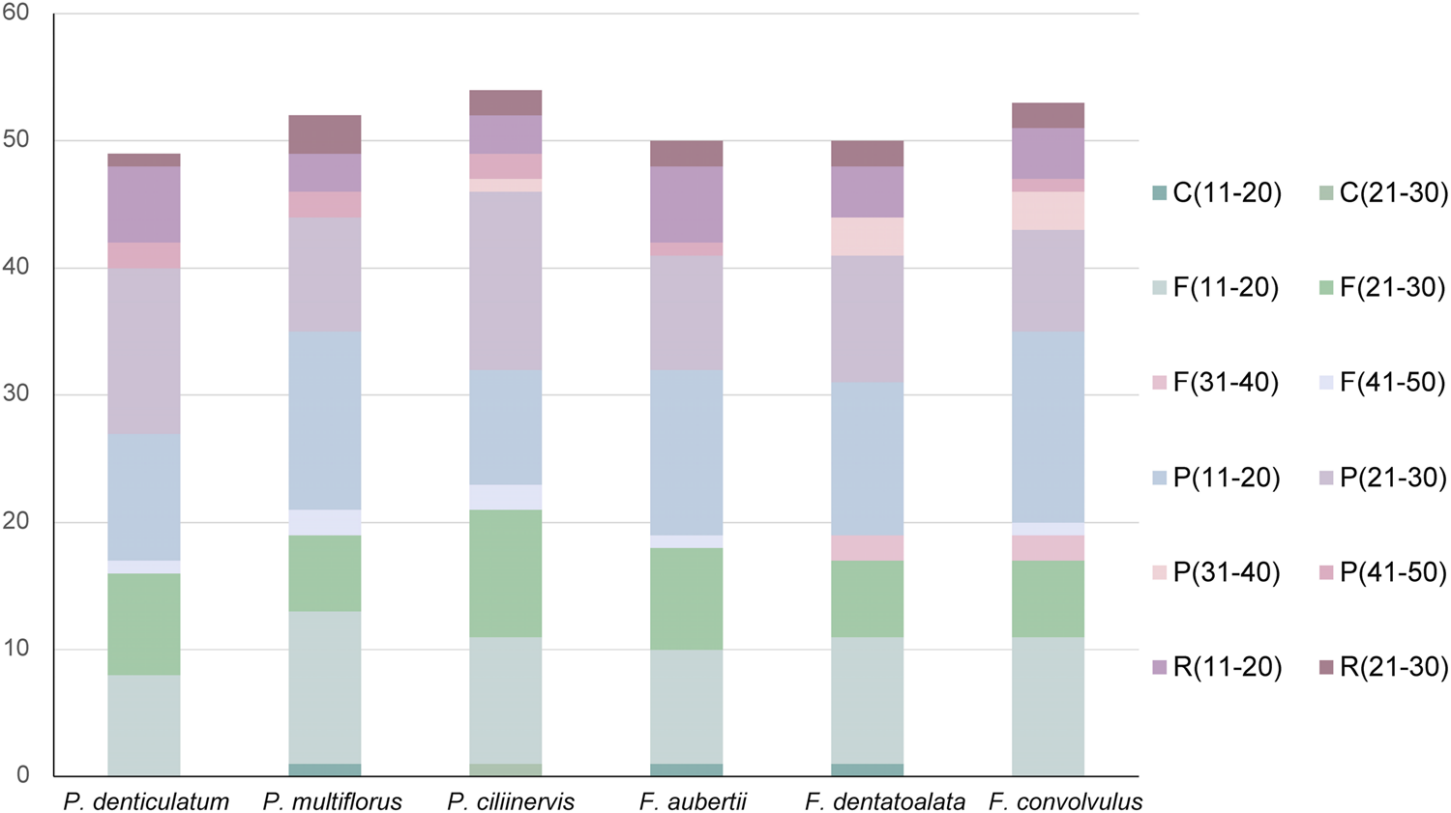
Types and numbers of repeat sequences in the chloroplast genomes of six species of Subfam. Polygonoideae. Note: F: forward repeat, R: inverted repeat, C: complementary repeat, P: palindromic repeat.

To identify the presence of long repeat sequences in the chloroplast genomes, the online tool REPuter was employed, and the distribution of these repeats is illustrated in Figs. 7. Interestingly, *P. denticulatum* and *F. convolvulus* lacked complement repeats in comparison to the other species. Among the six chloroplast genomes, palindrome repeats were identified, with the highest number falling within the range of 11–20 bp and 21–30 bp. Furthermore, no sequences exceeding 30 bp exhibited complement or reverse repeats, and there were no sequences surpassing 50 bp with forward or palindrome repeats.

### Phylogenetic analysis

**Figure 8:**
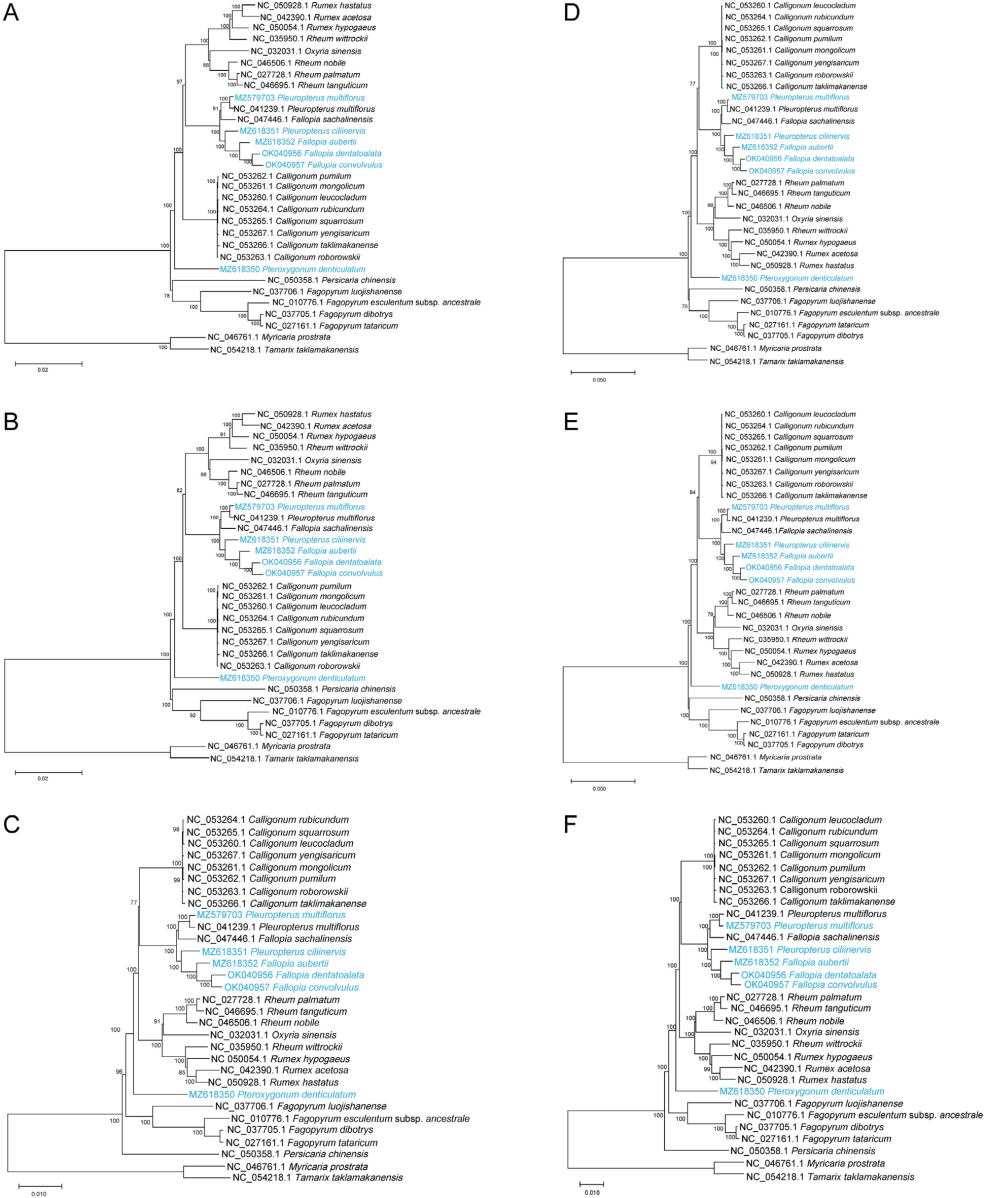
Exploration on the phylogeny of 29 species of Polygonaceae. (**A**) ML phylogenetic tree based on CDS sequence; (**B**) MP phylogenetic tree based on CDS sequence; (**C**) NJ phylogenetic tree based on CDS sequence; (**D**) ML phylogenetic tree based on complete chloroplast genome; (**E**) Based on MP phylogenetic tree of the complete chloroplast genome; (**F**) NJ phylogenetic tree based on the complete chloroplast genome.

To elucidate the phylogenetic position of the six Polygonaceae plants under investigation, a comprehensive phylogenetic analysis was conducted using 31 known chloroplast genome sequences from the Polygonaceae family. Both CDS and complete chloroplast genome sequences were employed in the phylogenetic analyses, and the results are presented in Figs. 8. The ML, MP, and NJ trees based on both CDS and complete chloroplast genome sequences exhibited strong branch support, indicating the reliability of the constructed trees. Significantly, *P. multiflorus* forms a cluster along with the NCBI publication of *P. multiflorus* (NC_041239.1), indicating a close relationship with *Fallopia sachalinensis*. Furthermore, *P. ciliinervis*, *F. aubertii*, *F. dentatoalata*, and *F. convolvulus* group together, while *P. denticulatum* holds a distinctive position outside this cluster, implying its divergence from the other five species.

### Selective pressures analysis

The non-synonymous to synonymous substitution ratio (ω = dN/dS) was examined for all 62 shared protein-coding genes across 31 complete chloroplast genomes in Polygonaceae. According to the M8 model (β & ω > 1), a total of 14 protein-coding genes exhibited positive selection with a posterior probability exceeding 0.95, as determined by the Bayes empirical Bayes (BEB) method (Table 3). Among these genes, *psbB* displayed the highest count of positive amino acid sites (74), trailed by *ycf1* (18) and *ycf2* (12). The remaining 11 protein-coding genes—namely *atpA*, *atpB*, *atpI*, *ndhA*, *ndhB*, *ndhF*, *ndhK*, *psbJ*, *rbcL*, *rpoA*, and *rps7*—each featured 2, 1, 1, 3, 4, 1, 1, 1, 3, 1, and 1 amino acid sites, respectively, that were conclusively under positive selection.

**Table 3:** Positive selective amino acid loci and estimation of parameters.

Gene	Ln L	Estimates of parameters	Positively selected sites
*atpA*	−4336.289477	p0 = 0.95824 p = 0.04328 q = 0.37472 (p1 = 0.04176) ω = 1.00000	465 F 0.972*, 465 F 0.972*
*atpB*	−4283.521151	p0 = 0.97318 p = 0.22341 q = 3.64282 (p1 = 0.02682) ω = 1.29802	18 K 0.982*
*atpI*	−2013.840531	p0 = 0.98099 p = 0.04230 q = 0.32409 (p1 = 0.01901) ω = 2.30130	12 K 0.987*
*ndhA*	−3444.921752	p0 = 0.91642 p = 5.59136 q = 99.00000 (p1 = 0.08358) ω = 1.24809	6 L 0.961*, 67 K 0.991**, 252 F 0.984*
*ndhB*	−4776.388234	p0 = 0.88005 p = 0.00500 q = 2.23523 (p1 = 0.11995) ω = 5.38061	17 R 0.956*, 228 V 1.000**, 259 W 0.957*, 836 P 0.992**
*ndhF*	−9590.248623	p0 = 0.94911 p = 0.28949 q = 1.13163 (p1 = 0.05089) ω = 1.90138	289 Y 0.969*
*ndhK*	−1904.101562	p0 = 0.97098 p = 0.03900 q = 0.22646 (p1 = 0.02902) ω = 2.17703	1 M 0.975*
*psbB*	−6077.589307	p0 = 0.49650 p = 0.05180 q = 0.07013 (p1 = 0.50350) ω = 5.10465	2 T 0.992**,3 A 0.992**,4 I 0.967*,6 E 1.000**,8 K 0.991**,9 A 0.981*,12 Y 0.991**,15 G 0.991**,17 H 1.000**,18 K 1.000**,20 S 0.969*,21 V 1.000**,25 V 1.000**,31 L 0.977*,32 L 0.983*,40 P 1.000**,45 N 0.993**,52 T 0.996**,61 F 1.000**,62 L 0.972*,64 Q 1.000**,66 V 0.994**,71 R 1.000**,72 Y 1.000**,81 R 1.000**,85 N 0.998**,88 H 1.000**,97 S 0.981*,104 W 1.000**,106 P 1.000**,111 H 0.999**,116 Q 1.000**,118 L 1.000**,123 I 0.979*,124 R 1.000**,130 P 1.000**,132 V 0.988*,137 V 0.997**,144 L 0.998**,145 T 0.995**,146 T 0.995**,147 S 0.996**,149 C 1.000**,150 T 0.950*,151 H 0.997**,154 C 0.999**,162 G 1.000**,169 G 0.999**,179 N 0.962*,182 M 0.969*,183 K 1.000**,184 S 0.963*,187 K 0.999**,190 T 0.990**,195 W 1.000**,197 F 0.998**,202 L 0.997**,204 S 0.970*,208 V 0.961*,215 G 1.000**,218 L 1.000**,222 T 0.995**,229 V 0.999**,234 L 0.998**,235 T 0.963*,236 S 0.960*,239 V 0.998**,241 T 1.000**,247 H 0.979*,248 E 0.998**,252 H 0.996**,257 A 0.994**,259 I 0.995**,265 N 0.996**
*psbJ*	−259.429719	p0 = 0.93917 p = 0.05072 q = 0.59102 (p1 = 0.06083) ω = 3.15524	25 I 0.966*
*rbcL*	−3721.582175	p0 = 0.93341 p = 0.04828 q = 0.66867 (p1 = 0.06659) ω = 1.27210	86 D 0.977*,230 C 0.985*,320 M 0.972*
*rpoA*	−2593.297147	p0 = 0.97440 p = 0.35912 q = 1.33619 (p1 = 0.02560) ω = 2.61713	251 F 0.959*
*rps7*	−2077.452951	p0 = 0.97103 p = 0.07168 q = 0.06340 (p1 = 0.02897) ω = 732.25016	5 C 0.992**
*ycf1*	−11,906.262213	p0 = 0.95677 p = 0.04133 q = 0.01998 (p1 = 0.04323) ω = 7.74282	387 F 0.987*,463 K 1.000**,464 I 1.000**,466 F 0.996**,587 G 0.959*,597 G 0.956*,754 Y 0.999**,827 F 0.999**,893 Y 0.996**,906 F 0.998**,933 Q 0.995**,935 K 0.973*,936 K 1.000**,937 F 0.996**,1093 F 0.997**,1127 S 0.985*,1248 Y 0.972*,1292 I 0.964*
*ycf2*	−27,710.541815	p0 = 0.96443 p = 0.21886 q = 0.05648 (p1 = 0.03557) ω = 6.95948	160 W 1.000**,513 V 0.955*,571 F 0.992**,629 D 1.000**,705 F 0.995**,1180 S 0.981*,1397 A 0.999**,2499 G 0.999**,3192 I 0.999**,3221 F 0.953*,3225 L 0.998**,3249 K 0.993**

* and ** indicate posterior probability higher than 0.95 and 0.99, respectively.

### Analysis of highly variable areas

**Figure 9:**
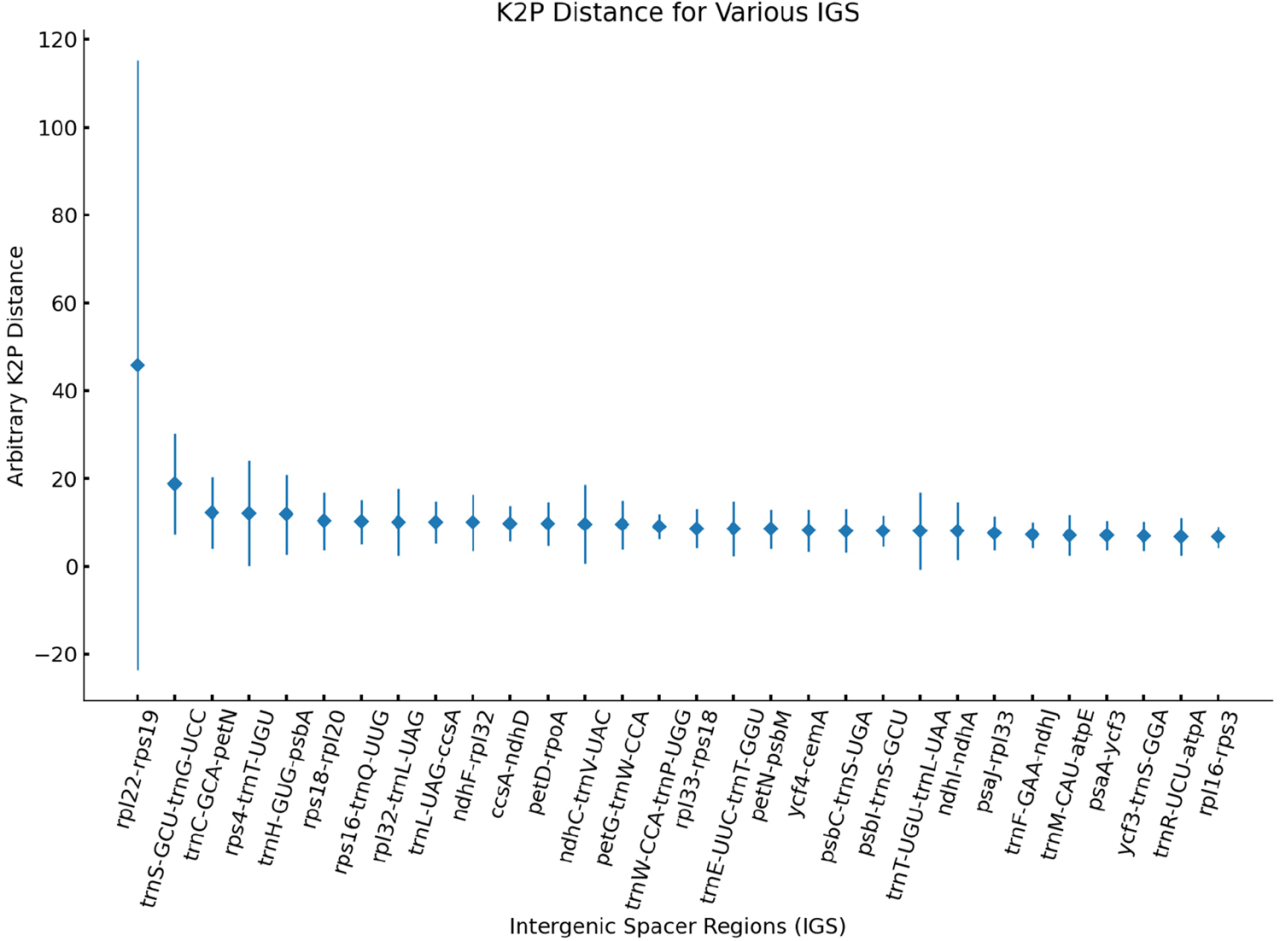
Analysis of hypervariable regions of chloroplast genomes of six species of Subfam. Polygonoideae.

In this study, we employed the K2p model to compare pairwise intergenic regions (IGS) and identify regions of pronounced differentiation among the six species (Fig. 9). Out of the 115 intergenic regions analyzed, the K2p genetic distances ranged from 0.24 to 81.3. Notably, the IGS regions such as *psaI-ycf4*, *rpl22-rps19*, and *trnS-GCU-trnG-UCC* exhibited the highest K2p genetic distances of 81.3, 52.25, and 20.25, respectively. These intergenic regions hold significant potential for the development of molecular markers in future studies.

### Primer design and PCR validation

**Figure 10:**
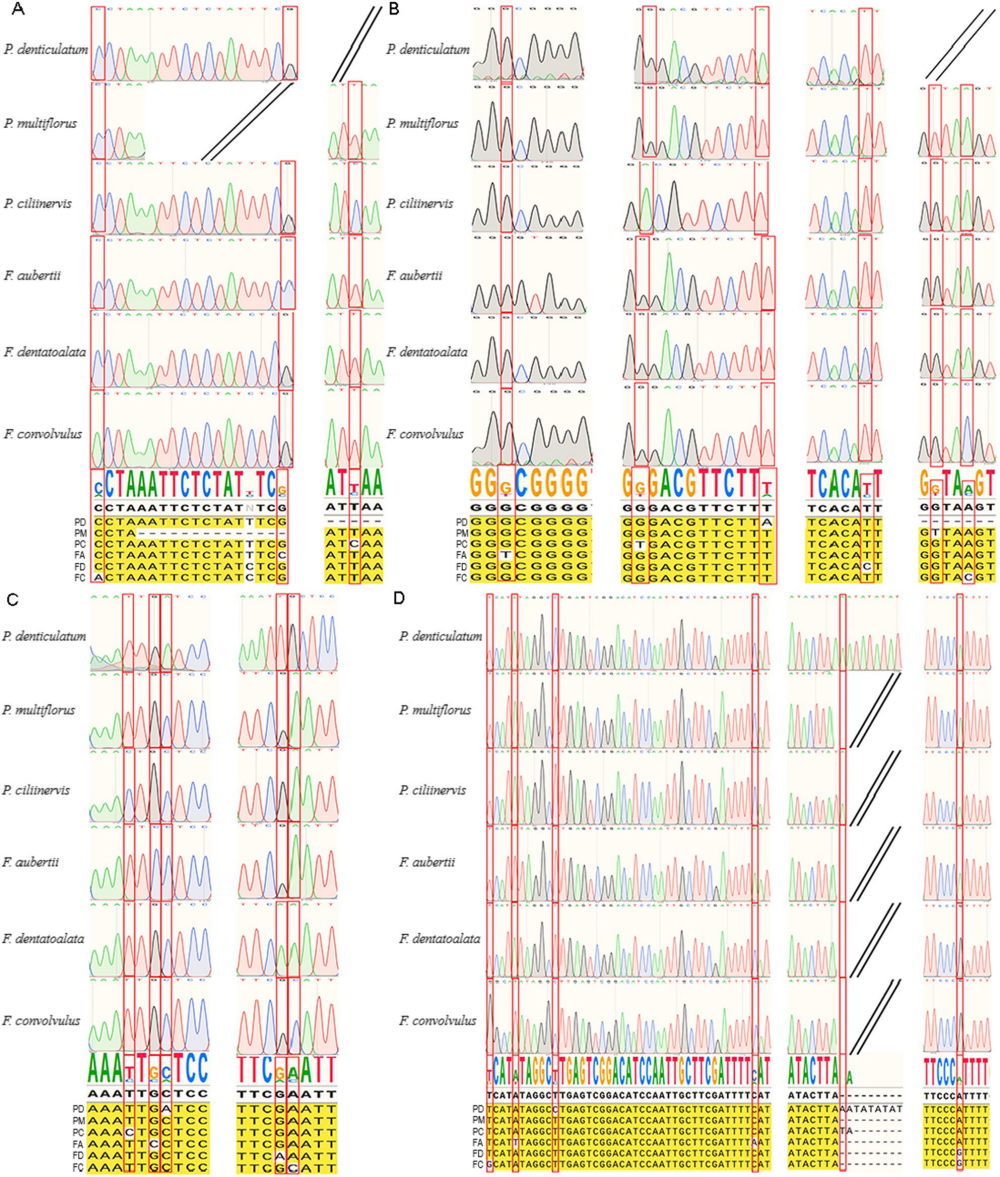
Alignment of sequencing chromatograms of PCR products. (**A**) petN-psbM primer; (**B**) psaI-ycf4 primer; (**C**) ycf3-trnS-GGA primer; (**D**) trnL-UAG-ccsA.

In order to differentiate among the six species, we employed SnapGene to screen 115 regions known for high variability. From this analysis, we identified 16 intergenic regions that could potentially serve as molecular markers (Table S4). PCR amplification of total DNA extracted from samples of the six species confirmed the specificity of *petN-psbM*, *psal-ycf4*, *ycf3-trnS-GGA*, and *trnL-UAG-ccsA* to these six species, yielding distinct and well-defined bands on agarose gels (Fig. S1). Subsequently, DNA fragments were extracted from each strip and subjected to Sanger sequencing, revealing variant loci among the different plants (Fig. 10). As a result, these four molecular markers, either individually or in combination, facilitate the identification of the six plants based on these variant loci.

## Disscussion

This study aimed to sequence and analyze the chloroplast genomes of six species belonging to Subfam. Polygonoideae. For the first time, we obtained the complete chloroplast genomes of *P. denticulatum*, *F. aubertii*, *F. dentatoalata*, and *F. convolvulus*. The chloroplast genomes of these six species exhibit a typical quadripartite structure, which is consistent with other plants of the Polygonaceae family, such as *Calligonum* L.^48^, *Polygonum* L.^23^, *F. sachalinensis*^49^, and *Reynoutria japonica* Houtt.^24^. This phenomenon is also widely observed in other angiosperm chloroplast genomes^50–52^.

The lengths of the chloroplast genomes in these six species do not differ significantly, and the lengths of the tetrad regions are consistent with other Polygonaceae plants, including *Fagopyrum* Mill.^53^, *Muehlenbeckia* Meisn.^54^, and *Persicaria chinensis* (L.) H. Gross^21^. Furthermore, the GC content of the chloroplast genomes in these six plants is lower than the AT content, which is also observed in other Polygonaceae plants such as *Fagopyrum dibotrys* (D. Don) Hara^55^ and *R. japonica*^56^. The higher GC content in the IR region compared to the LSC and SSC regions is worth noting, possibly attributed to the abundance of rRNA within the IR region. These aforementioned plants belong to distinct genera within the Polygonaceae classification, and their geographical distribution is scattered. This indicates that the chloroplast genomes of most Polygonaceae plants share common structural and length characteristics.

Introns, non-coding regions found in RNA transcripts or DNA encoding RNA, play a vital role in gene expression regulation^57^. They can significantly enhance the expression of exogenous genes at specific times and locations in plants, thereby controlling gene expression levels across various spatial and temporal contexts^58^. In our study, we identified genes with introns in all six plant species examined, with the number of introns ranging from 18 to 21. Notably, we observed a consistent pattern wherein both *clpP* and *ycf3* genes in each plant contained two introns and three exons. This finding differs from the results reported by Guo et al.^23^ for *Polygonum*, which also belongs to the Polygonaceae family. Guo et al. showed that four *Polygonum* plants possessed only one gene (*rps12*) with three exons, while two genes (*ycf3* and *clpP*) contained two introns. Furthermore, they observed that the 5' end of the *rps12* gene in *Polygonum* was located in the LSC region, with its 3' end duplicated in the IRs region, whereas in all plants examined in our study, the *rps12* gene was exclusively situated in the IR region. Although the occurrence and loss of introns in plant chloroplast genomes are observable, the precise regulatory mechanisms governing them in plants remain unclear^23^. Moreover, there exists a research gap concerning the study of introns in Polygonaceae plants, highlighting the need for future investigations to gain a deeper understanding of their functions.

The presence of the IR region is a recurring feature in most chloroplast genomes^16^, and the size of the IR region, containing rRNA genes, varies significantly across different biological clades^59^. Four junctions, namely JLA, JSA, JSB, and JLB, exist between the two IRs and the SC region^60^. The successive expansions of the IR have led to floating JLAs and JLBs, which hold evolutionary significance^61^. However, the mechanism behind IR region expansion and contraction, primarily governed by the double-strand break repair (DCBR) theory^62^, remains a subject of controversy. In our study, we observed similar levels of IR boundary contraction and expansion among all six Polygonoidea plants. Specifically, *rps19* and *ndhF* straddle the JLB and JSB boundaries, respectively, while *ycf1* is exclusively present in the IRa region. This consistency aligns with *Polygonum*^23^. Based on these findings, it is reasonable to speculate that all or some of the biological lineages within Polygonoidea share the same IR boundary feature. To confirm this hypothesis, further analysis of chloroplast genomes in Polygonoidea plants is warranted.

Codons, also known as the genetic code, serve as a crucial link between nucleic acids and proteins. They play a pivotal role in recognizing and transmitting biological genetic information, contributing significantly to genetic processes and variations in living organisms^63^. Due to variations in protein translation processes across different species, there is a tendency to utilize specific synonymous codons during translation, which is referred to as codon usage bias (CUB)^64^, which plays an important role in cellular metabolic processes such as mRNA translation and DNA transcription^65^. In the chloroplast genomes of the six plants examined in this study, Arg and Leu were found to be more frequently used. Among amino acids with a total codon count below 2,000, Cys exhibited the lowest frequency, aligning with patterns observed in terrestrial plants like *Allium mongolicum* Regel^66^ and *Psammosilene tunicoides* W.C. Wu et C.Y. Wu^67^. All six species displayed a preference for UAA as a termination codon, and each species exhibited 30 codons with RSCU values greater than 1. Remarkably, 29 out of these 30 codons concluded with A/U, demonstrating strong consistency with the observed phenomenon in *Polygonum*, which also belongs to Polygonoidea^23^.

Variable regions of the chloroplast genome are usually associated with many repetitive sequences, and most of them are located in intergenic regions or on the same gene^68^. Simple repeat sequences (SSRs), also known as Microsatellites, are widely distributed in chloroplast genomes and are extremely variable, and therefore are often used as molecular markers to study chloroplast genome evolution and population genetics^69,70^. In the six selected plant chloroplast genomes analyzed in this study, the SSRs primarily consist of single nucleotides, aligning with observations in *Polygonum*. However, it is noteworthy that the total number of SSRs in the studied plants is significantly lower than that observed in *Polygonum*. Moreover, highly variable regions play a crucial role in resolving phylogenies and distinguishing closely related plant species^71^. In this study, we employed a combined approach of highly variable region analysis and PCR amplification analysis to identify four chloroplast gene regions suitable as molecular markers, namely *petN-psbM*, *psal-ycf4*, *ycf3-trnS-GGA*, and *trnL-UAG-ccsA*. Single-nucleotide polymorphisms within intergenic regions may directly impact the structural conformation or expression levels of proteins, depending on their specific locations. This could potentially influence the morphology and genetic mechanisms of plants^72^. Prior research has successfully employed molecular markers derived from *petN-psbM* to differentiate between five *Alpinia* Roxb. species^73^. The variable hotspot region of *ycf3-trnS-GGA* has been utilized for assessing interspecific differentiation in Dipsacales species. It is proposed as a candidate DNA barcode for species within Adoxaceae and Caprifoliaceae^74^. Similarly, this region has been employed for identifying three *Salvia* L. species^75^.

Previous studies have utilized chloroplast genomic techniques to conduct phylogenetic analysis of Polygonaceae plants, shedding light on the issue of taxonomic confusion within this plant family^6,23,24^. In this study, we employed both CDS and whole genome data to reconstruct the phylogenetic relationships among 29 Polygonaceae plants. Remarkably, the phylogenetic trees constructed using these two data types exhibited high consistency, validating the reliability of utilizing chloroplast genomes for phylogenetic reconstruction. Notably, our findings revealed a distinct clustering of *F. sachalinensis* with *P. multiflorus* within the same clade, while *P. ciliinervis* displayed a closer relationship with Genus *Fallopia*. These results diverge from the current classification wherein *P. multiflorus* and *P. ciliinervis* are grouped within the same genus^7^. Additionally, our results consistently support the conclusion that *P. denticulatum* does not belong to Genus *Fallopia* and that *F. dentatoalata* is more closely related to *F. convolvulus* in Genus *Fallopia*, aligning with previous studies^6,76^. However, due to the unavailability of fresh leaves from *P. giraldii* and the subsequent lack of chloroplast genomic data for this species, we were unable to ascertain the phylogenetic relationship between *P. denticulatum* and *P. giraldii* based on chloroplast genomes. This calls for more comprehensive sampling and further investigation in future studies.

Positive selection is considered pivotal in the adaptation of organisms to various environments^77^, while negative (purifying) selection stands as a prevalent evolutionary force accountable for genomic sequence conservation over extensive evolutionary periods^78^. The ratio (ω = dN/dS) has served as a widely employed metric for gauging selective pressure^79–81^. The ω ratio > 1 represents positive selection, whereas ω < 1 indicates purifying selection^80^. In this investigation, we identified 14 genes harboring positive selection sites. Within these genes featuring amino acid positive sites, it was observed that the *psbB*, *ycf1*, and *ycf2* genes exhibited a higher count (74, 18, 12 respectively) of positive amino acid sites among Polygonaceae species, suggesting a potentially significant role of the *psbB* gene in the adaptive evolution of Polygonaceae species. Additionally, a gene (*rps7*) encoding ribosomal subunit protein was found under positive selection, which is deemed crucial for chloroplast biogenesis and function, implying that Polygonaceae plants might enhance evolutionary adaptability by modulating the encoding of ribosomal subunit protein in chloroplasts^82^. Furthermore, nine photosynthesis-related genes, namely *atpA*, *atpB*, *atpI*, *psbB*, *psbJ*, *ndhA*, *ndhB*, *ndhF*, *ndhk*, and *rbcL*, were also identified with positive selection sites in the present study. Recent investigations have indicated the prevalence of these 14 genes exhibiting positive selection across certain angiosperms. For instance, *ndhF* has been documented as undergoing positive selection in Aroideae species^79^; *ndhA*, *ndhB*, *psbB*, *rbcL*, *rps7*, *ycf1*, and *ycf2* have been reported as undergoing positive selection in *Zingiber* species^83,84^; while *atpA*, *atpB*, *atpI* have been identified as undergoing positive selection in *Chrysosplenium* species^85^. Polygonaceae represents a cosmopolitan plant family widely distributed across the northern temperate regions and occasionally found in tropical regions^86^. Consequently, Polygonaceae species are likely subjected to various environmental stresses in their ecological habitats, and these 14 genes undergoing positive selection may exert crucial roles during the evolution and adaptation of Polygonaceae species to their respective ecological habitats.

Polygonaceae encompasses a diverse array of medicinal and horticultural plants that possess significant economic value^6,87^. However, certain species within the Polygonaceae family exhibit a highly invasive nature in certain countries and regions, leading to severe ecological damage^5^. The absence of a robust taxonomy, grounded in an understanding of phylogenetic relationships, poses obstacles to the advancement of cash crops within Polygonaceae and the effective management of invasive species. Thus, it is imperative to undertake comprehensive investigations into the phylogeny of Polygonaceae plants in order to address these concerns. This paper focuses on three genera within Subfam. Polygonoidea, namely Pteroxygonum, Pleuropterus, and *Fallopia*. For the first time, we have obtained the complete chloroplast genome data of *P. denticulatum*, *P. ciliinervis*, *F. aubertii*, *F. dentatoalata,* and *F. convolvulus*. These newly acquired chloroplast genome data serve as a valuable resource for understanding the taxonomic, phylogenetic, and evolutionary history of Polygonoidea.

### Supplementary Information

Supplementary Information.

## Data Availability

The complete chloroplast genomes were generated in the NCBI database with Accession Number MZ618350, MZ579703, MZ618351, MZ618352, OK040956, OK040957.
