# Supplementary material for: Phylogenetic relationships, selective pressure and molecular markers development of six species in subfamily Polygonoideae based on complete chloroplast genomes
Source: Sci Rep. 2024 Apr 29;14:9783. doi: 10.1038/s41598-024-58934-7 (PMC11059183; doi:10.1038/s41598-024-58934-7)
Supplement: Supplementary file 1 — Supplementary Information. [file 41598_2024_58934_MOESM1_ESM.pdf]

Phylogenetic Relationships, Selective Pressure and Molecular Markers Development of six species in Subfamily Polygonoideae Based on Complete Chloroplast Genomes

Zhan Feng1,2#, Yan Zheng1,3#, Yuan Jiang1,4, Jin Pei2,5*, Linfang Huang1*

1 Key Laboratory of Chinese Medicine Resources Conservation, State Administration of Traditional Chinese Medicine of the People's Republic of China, Institute of Medicinal Plant Development, Chinese Academy of Medical Sciences & Peking Union Medical College, Beijing, China

2 College of Pharmacy, Chengdu University of Traditional Chinese Medicine, Chengdu, Sichuan, China

3 Jiangxi University of Chinese Medicine, Nanchang, Jiangxi, China

4 Beijing University of Chinese Medicine, Beijing, China

5 State Key Laboratory of Southwestern Chinese Medicine Resources, Chengdu University of Traditional Chinese Medicine, Chengdu, Sichuan, China

#: Zhang Feng and Yan Zheng: These authors contributed equally to this work and share first authorship.

*Correspondence:

Jin Pei: peixjin@163.com

Linfang Huang: lfhuang@implad.ac.cn

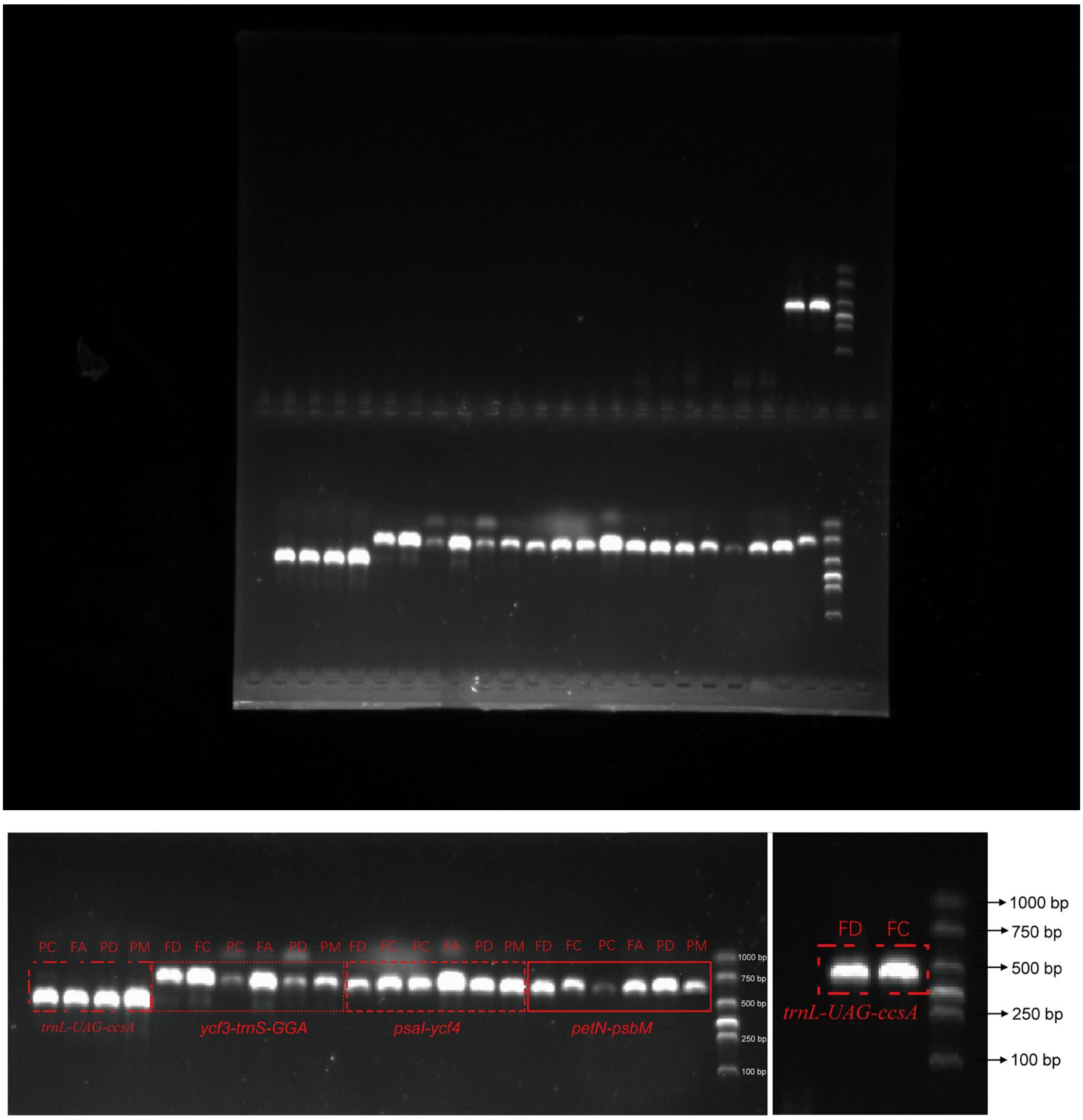

Figure S1. Gel electrophoresis results of amplification of DNA barcodes using designed primers. PD: *P. denticulatum*, PM: *P. multiflorus*, PC: *P. ciliinervis*, FA: *F. aubertii*, FD: *F. dentatoalata*, FC: *F. convolvulus*.

Table S1. Statistics of sequencing results of the chloroplast genomes of six plants of Subfam. Polygonoideae.

Species	Raw reads (bp)	Clean reads (bp)	Raw base (G)	Clean base (G)
P. denticulatum	19,112,049	19,013,128	5.73	5.7
P. multiflorus	17,979,597	17,924,381	5.39	5.38
P. ciliinervis	17,329,101	17,280,207	5.2	5.18
F. aubertii	21,319,251	21,239,370	6.4	6.37
F. dentatoalata	18,831,608	18,796,229	2.82	2.82
F. convolvulus	18,508,639	18,470,144	2.77	2.77

Table S2. Basic characteristics of chloroplast genomes of six species of Polygonoideae.

Species	Region	Length of the genome /bp	GC	A	T	C	G
P. denticulatum	Total	162,928	37.38%	31.17%	31.46%	19.01%	18.37%
	IRa	30,853	41.24%	28.63%	30.13%	21.50%	19.74%
	IRb	30,853	41.24%	30.13%	28.63%	19.74%	21.50%
	SSC	13,170	32.57%	36.06%	31.37%	17.06%	15.51%
	LSC	88,052	35.39%	31.68%	32.93%	18.17%	17.22%
P. multiflorus	Total	163,425	37.54%	31.09%	31.37%	19.07%	18.47%
	IRa	30,899	41.25%	28.60%	30.15%	21.59%	19.66%
	IRb	30,899	41.25%	30.15%	28.60%	19.66%	21.59%
	SSC	13,509	32.82%	35.43%	31.76%	17.11%	15.70%
	LSC	88,118	35.66%	31.63%	32.72%	18.27%	17.39%
P. ciliinervis	Total	163,583	37.36%	31.17%	31.47%	19.02%	18.34%
	IRa	30,887	41.21%	28.65%	30.15%	21.55%	19.66%
	IRb	30,887	41.21%	30.15%	28.65%	19.66%	21.55%
	SSC	13,564	32.28%	35.73%	31.99%	16.93%	15.35%
	LSC	88,245	35.44%	31.71%	32.85%	18.23%	17.21%
F. aubertii	Total	162,393	37.59%	31.05%	31.37%	19.10%	18.49%
	IRa	30,860	41.24%	28.64%	30.12%	21.55%	19.70%
	IRb	30,860	41.24%	30.12%	28.64%	19.70%	21.55%
	SSC	13,394	32.78%	35.52%	31.70%	17.14%	15.63%
	LSC	87,279	35.74%	31.54%	32.72%	18.32%	17.42%
F. dentatoalata	Total	162,625	37.61%	31.03%	31.37%	19.12%	18.49%
	IRa	30,868	41.27%	28.62%	30.12%	21.54%	19.73%
	IRb	30,868	41.27%	30.12%	28.62%	19.73%	21.54%
	SSC	13,549	32.70%	35.71%	31.58%	17.20%	15.51%
	LSC	87,340	35.78%	31.47%	32.75%	18.35%	17.43%
F. convolvulus	Total	162,636	37.66%	31.01%	31.33%	19.14%	18.52%
	IRa	30,876	41.28%	28.60%	30.11%	21.55%	19.73%
	IRb	30,876	41.28%	30.11%	28.60%	19.73%	21.55%
	SSC	13,537	32.92%	35.63%	31.45%	17.28%	15.65%
	LSC	87,347	35.83%	31.47%	32.70%	18.37%	17.47%

Table S3. Length of exons and introns in six chloroplast genomes of Polygonoideae.

Specises	Gene	Strand	Start	End	ExonI	IntronI	ExonII	IntronII	ExonIII
	rnK-UUU	-	1713	4312	37	2528	35		
	rps16	-	5202	6249	41	797	210		
	trnG-UCC	+	9868	10612	23	674	48		
	atpF	-	12542	13857	145	761	410		
	rpoC1	-	21417	24235	432	776	1611		
	ycf3	-	44837	46815	124	729	230	743	153
	trnL-UAA	+	50531	51135	37	518	50		
	trnV-UAC	-	54946	55596	38	578	35		
	clpP	-	73729	75924	71	945	291	618	271
	petB	+	78865	80235	6	723	642		
P. denticulatum	*petD*	+	80447	81720	8	791	475		
	rpl16	-	85146	86621	9	1068	399		
	ndhB	-	98464	100675	775	679	758		
	trnI-GAU	+	106024	107041	37	946	35		
	trnA-UGC	+	107106	107970	38	792	35		
	ycf1	+	113020	118656	611	33	4993		
	ndhA	-	128371	130499	553	1037	539		
	ycf1	-	132325	137961	611	33	4993		
	trnA-UGC	-	143011	143875	38	792	35		
	trnI-GAU	-	143940	144957	37	946	35		
	ndhB	+	150306	152517	775	679	758		
	trnK-UUU	-	1687	4263	37	2505	35		
	rps16	-	5227	6357	41	864	226		
	trnG-UCC	+	10311	11104	23	723	48		
	atpF	-	13051	14366	145	761	410		
	rpoC1	-	21870	24693	432	781	1611		
	ycf3	-	45314	47299	124	737	230	742	153
	trnL-UAA	+	50381	51032	37	565	50		
	trnV-UAC	-	54473	55130	38	585	35		
	clpP	-	73790	76113	71	1066	291	625	271
P. multiflorus	*petB*	+	79051	80425	6	727	642		
	petD	+	80661	81897	8	754	475		
	rpl16	-	85341	86675	9	927	399		
	ndhB	-	98492	100699	775	675	758		
	trnI-GAU	+	106081	107101	37	949	35		
	trnA-UGC	+	107168	108041	38	801	35		
	ndhA	-	128755	130946	553	1100	539		
	trnA-UGC	-	143503	144376	38	801	35		
	trnI-GAU	-	144443	145463	37	949	35		
	ndhB	+	150845	153052	775	675	758		
	trnK-UUU	-	1635	4215	37	2509	35		
	rps16	-	5160	6311	41	885	226		
	trnG-UCC	+	10445	11237	23	722	48		
	atpF	-	13202	14516	145	760	410		
	rpoC1	-	22043	24867	432	782	1611		
	ycf3	-	45374	47363	124	733	230	750	153
	trnL-UAA	+	50454	51147	37	607	50		
	trnV-UAC	-	54584	55238	38	582	35		
P. ciliinervis	*clpP*	-	73865	76175	71	1059	291	619	271
	petB	+	79116	80469	6	706	642		
	petD	+	80711	81950	8	757	475		
	ndhB	-	98606	100813	775	675	758		
	trnI-GAU	+	106197	107217	37	949	35		
	trnA-UGC	+	107284	108157	38	801	35		
	ndhA	-	128856	131095	553	1148	539		
	trnA-UGC	-	143672	144545	38	801	35		
	trnI-GAU	-	144612	145632	37	949	35		
	ndhB	+	151016	153223	775	675	758		
	trnK-UUU	-	1713	4312	37	2528	35		
	rps16	-	5202	6249	41	797	210		
	trnG-UCC	+	9868	10612	23	674	48		
	atpF	-	12542	13857	145	761	410		
	rpoC1	-	21417	24235	432	776	1611		
	ycf3	-	44837	46815	124	729	230	743	153
	trnL-UAA	+	50531	51135	37	518	50		
	trnV-UAC	-	54946	55596	38	578	35		
	clpP	-	73729	75924	71	945	291	618	271
	petB	+	78865	80235	6	723	642		
F. aubertii	*petD*	+	80447	81720	8	791	475		
	rpl16	-	85146	86621	9	1068	399		
	ndhB	-	98464	100675	775	679	758		
	trnI-GAU	+	106024	107041	37	946	35		
	trnA-UGC	+	107106	107970	38	792	35		
	ycf1	+	113020	118656	611	33	4993		
	ndhA	-	128371	130499	553	1037	539		
	ycf1	-	132325	137961	611	33	4993		
	trnA-UGC	-	143011	143875	38	792	35		
	trnI-GAU	-	143940	144957	37	946	35		
	ndhB	+	150306	152517	775	679	758		
	trnK-UUU	-	1587	4177	37	2519	35		
	rps16	-	5114	6254	41	874	226		
	trnG-UCC	+	9476	10258	23	712	48		
	atpF	-	12213	13526	145	759	410		
	rpoC1	-	21058	23884	432	784	1611		
	ycf3	-	44491	46483	124	733	230	753	153
	trnL-UAA	+	49613	50243	37	544	50		
	trnV-UAC	-	53675	54331	38	584	35		
	clpP	-	73077	75424	71	1085	291	630	271
F. dentatoalata	*petB*	+	78310	79727	6	770	642		
	petD	+	79968	81213	8	763	475		
	rpl16	-	84588	85897	9	902	399		
	ndhB	-	97707	99913	775	674	758		
	trnI-GAU	+	105300	106316	37	945	35		
	trnA-UGC	+	106383	107255	38	800	35		
	ndhA	-	127910	130169	553	1168	539		
	trnA-UGC	-	142711	143583	38	800	35		
	trnI-GAU	-	143650	144666	37	945	35		
	ndhB	+	150053	152259	775	674	758		
	trnK-UUU	-	1587	4173	37	2515	35		
	rps16	-	5064	6198	41	868	226		
	trnG-UCC	+	9428	10212	23	714	48		
	atpF	-	12165	13476	145	757	410		
	rpoC1	-	21010	23837	432	785	1611		
	ycf3	-	44478	46463	124	732	230	747	153
	trnL-UAA	+	49578	50197	37	533	50		
	trnV-UAC	-	53637	54293	38	584	35		
	clpP	-	73058	75422	71	1098	291	634	271
F. convolvulus	*petB*	+	78369	79777	6	761	642		
	petD	+	80013	81260	8	765	475		
	rpl16	-	84634	85904	9	863	399		
	ndhB	-	97713	99919	775	674	758		
	trnI-GAU	+	105306	106324	37	947	35		
	trnA-UGC	+	106391	107263	38	800	35		
	ndhA	-	127942	130165	553	1132	539		
	trnA-UGC	-	142721	143593	38	800	35		
	trnI-GAU	-	143660	144678	37	947	35		
	ndhB	+	150065	152271	775	674	758		

Table S4. Primers designed for six chloroplast genome identifications using highly variable regions.

ID	Region	Type of	Conserved Sequences,5'-3'
1	*matK-rps16*	Forward	TTTGAATTCATTTCGTTTCGTTCTAAG
		Reverse	GAGTTTGTAAGACCACGACTGA
2	*ndhF-rpl32*	Forward	ATTTTCGATTCACCGGCTCTT
		Reverse	TTTGGTCATTTGACCAATTAGAACTTCT
3	*petA-psbJ*	Forward	TTTGCCTTCCTAATCTTCGACA
		Reverse	AAGTTCATTGAAGAAGTTCTATTTGTTG
4	*petB-petD*	Forward	GAAGATAGATCATAGATCTTTGTAAT
		Reverse	AATCCATTTTCCTTTCGGAGAA
5	*petN-psbM*	Forward	AATAATGTCAATCAAACAGATATTTCAATGA
		Reverse	TTATGCGTAGTATCTTGTTGAAGAAC
6	*psaA-ycf3*	Forward	TGAGTCCTCCTCTTTCCGGA
		Reverse	TTCTTTTCGATTATGAGCAATTAATACCT
7	*psaI-ycf4*	Forward	TTTATTGGAAAGTGGAATATGGTATAATG
		Reverse	AATTGATAGAATAACCAAAATGAATTTCAC
8	*psbJ-psbF*	Forward	TGCTAGAGACATAAACAGTCATGG
		Reverse	ACAAAACCTAATCCGAATTATAGAGCT
9	*rpl20-clpP*	Forward	TGAATAAATGAAACTTTGCTAAATAACTAAT
		Reverse	GAGAGGACCTCACCGTTTAAG
10	*rpoB-trnC-GCA*	Forward	AATTGAATTTGTAAGAGAAGAGATGAAAG
		Reverse	TACTGTTTCTGATTATTCTATATTTATCTCG
11	*rps16-trnQ-UUG*	Forward	ATTATGGAATCATGAATAGTCATTGGTT
		Reverse	ATCCCTTAAATGAGGCAGCTAAATTATAA
12	*trnE-UUC-trnT-GGU*	Forward	TATCAACAGTTTTTCGAAATTGTCAA
		Reverse	ACTATCACTTCAATGAACCAAGC
13	*trnN-GUU-trnR-ACG*	Forward	CTCATCGTATGACATTCTGTTCT
		Reverse	TTTTACGTCCCGTAACTCTTC
14	*trnR-ACG-trnN-GUU*	Forward	GAGCTCGAAGCTTCCTTCG
		Reverse	TTATATAATAAGTTTTTACCTTCTTATTATAAGATAATC
15	*ycf3-trnS-GGA*	Forward	CAAAATTGCATAAAAAAGATAAAAAAAGAAAT
		Reverse	GATTCTTTTTCCCTCCCTCTT
16	*trnL-UAG-ccsA*	Forward	ATCTTGAAAGTGATTCGTATTCCAT
		Reverse	GAGTCTATTTCATCCCTCTTACTAAC
